# *Anais Brasileiros de Dermatologia*: new challenges. New guidelines for authors^☆^

**DOI:** 10.1016/j.abd.2021.02.001

**Published:** 2021-03-20

**Authors:** Silvio Alencar Marques, Ana Maria Roselino, Hiram Larangeira de Almeida, Luciana P. Fernandes Abbade

**Affiliations:** a Universidade Estadual Paulista, Botucatu, SP, Brazil; b Universidade de São Paulo, Ribeirão Preto, SP, Brazil; c Universidade Federal de Pelotas, Pelotas, RS, Brazil; d Universidade Católica de Pelotas, Pelotas, RS, Brazil

Dear colleagues,

As stated in the Editorial of the previous issue of the *Anais Brasileiros de Dermatologia* (ABD),^1^ one of our greatest challenges is to increase the scientific quality of the journal as much as possible, aiming to make it more visible and appreciated by national and international authors. For that purpose, in addition to everyone’s invaluable efforts, particularly those who submit their articles to ABD, some adjustments to the Guidelines for Authors are necessary. Much has been debated by the previous editors and reiterated by the current management concerning the number of authors in the articles in different sections, with emphasis in the section now called “Original Articles”, which has replaced the previously called “Investigation” section. It was decided to eliminate the restriction on the number of authors in the “Original Articles” section, recognizing the complexity of articles that require multiple efforts, for instance, a multidisciplinary team or a multicenter study setting. Also, it was decided to increase the number allowed in several other sections. The commitment to list as authors those who were actually part of the article creation and share the responsibility for it is emphasized. In addition to this modification, certain specifications to be met by the authors of the studies were included in well-defined fields, namely: for reports of randomized clinical trials, they must follow the CONSORT recommendations (http://www.consort-statement.org/consort-statement/checklist);^2^ for the reporting of a systematic review with or without meta-analysis, which requires specific methodologies, they must follow the PRISMA recommendations (http://prisma-statement.org/prismastatement/Checklist.aspx).^3^ Observational studies, in turn, widely used in Dermatology, must be described and specified according to the STROBE recommendations (https://www.strobe-statement.org/index.php?id=available-checklists).^4^ Also, series of cases, which can be submitted as “Original Articles” are reported according to the CARE checklist (https://www.care-statement.org/checklist). Articles that do not meet the profile of those mentioned above can be viewed and are reported on the EQUATOR website (http://www.equator-network.org/).

In addition to these changes, which will certainly require the authors’ qualification and commitment, there are new instructions regarding other sections and the Research Ethics Commission’s protocol. As for the illustrations, special consideration is requested for the illustrations in the Dermatopathology section.

We invite the readers to access the ABD website at http://www.anaisdedermatologia.org.br/ to get acquainted with these new guidelines.

Finally, to make it easier for the authors, the Consent to Use in Publication and the Conflict of Interest Declaration forms have been modified, in which signatures are now restricted to a single document and are entirely presented online.

Accordingly, we are on this path together, in search of increasing qualification, which will result in an increase in the Impact Factor for ABD, in line with the quality of the Ibero-Latin American Dermatology.

## Financial support

None declared.

## Authors’ contributions

Silvio Alencar Marques: Approval of the final version of the manuscript; preparation and writing of the manuscript.

Ana Maria Roselino: Approval of the final version of the manuscript; preparation and writing of the manuscript.

Hiram Larangeira de Almeida Junior: Approval of the final version of the manuscript; preparation and writing of the manuscript.

Luciana P. Fernandes Abbade: Approval of the final version of the manuscript; preparation and writing of the manuscript.

## Conflicts of interest

None declared.
